# In-Depth Characterization of Endo-Lysosomal Aβ in Intact Neurons

**DOI:** 10.3390/bios12080663

**Published:** 2022-08-20

**Authors:** Alec K. McKendell, Mei C. Q. Houser, Shane P. C. Mitchell, Michael S. Wolfe, Oksana Berezovska, Masato Maesako

**Affiliations:** 1 Alzheimer Research Unit, MassGeneral Institute for Neurodegenerative Disease, Massachusetts General Hospital, Harvard Medical School, 114, 16th street, Charlestown, MA 02129, USA; 2 Department of Medicinal Chemistry, University of Kansas, 1567 Irving Hill Rd, Lawrence, Kansas City, KS 66045, USA

**Keywords:** Alzheimer’s disease, fluorescence resonance energy transfer (FRET), γ-secretase, glycoconjugates, intracellular amyloid-beta (Aβ), lysosomes

## Abstract

Amyloid-beta (Aβ) peptides are produced within neurons. Some peptides are released into the brain parenchyma, while others are retained inside the neurons. However, the detection of intracellular Aβ remains a challenge since antibodies against Aβ capture Aβ and its precursor proteins (i.e., APP and C99). To overcome this drawback, we recently developed 1) the C99 720-670 biosensor for recording γ-secretase activity and 2) a unique multiplexed immunostaining platform that enables the selective detection of intracellular Aβ with subcellular resolution. Using these new assays, we showed that C99 is predominantly processed by γ-secretase in late endosomes and lysosomes, and intracellular Aβ is enriched in the same subcellular loci in intact neurons. However, the detailed properties of Aβ in the acidic compartments remain unclear. Here, we report using fluorescent lifetime imaging microscopy (FLIM) that intracellular Aβ includes both long Aβ intermediates bound to γ-secretase and short peptides dissociated from the protease complex. Surprisingly, our results also suggest that the dissociated Aβ is bound to the glycoproteins on the inner membrane of lysosomes. Furthermore, we show striking cell-to-cell heterogeneity in intracellular Aβ levels in primary neurons and APP transgenic mouse brains. These findings provide a basis for the further investigation of the role(s) of intracellular Aβ and its relevance to Alzheimer’s disease (AD).

## 1. Introduction

Alzheimer’s disease (AD) is a complex, progressive neurodegenerative disorder that causes memory loss and cognitive impairments. Currently clinically available drugs help lessen or stabilize cognitive symptoms [1,2,3,4]. In addition, combination therapy shows superior clinical outcomes [5,6,7]. Nevertheless, these drugs do not stop neurodegeneration; therefore, disease-modifying therapy is highly demanded. Senile plaques are one of two brain lesions used to diagnose AD. These aggregates primarily consist of amyloid-beta (Aβ) peptides 40 or 42 amino acids in length, though Aβ42 is more likely to aggregate and accumulate and is considered pathogenic [8,9,10]. Aβ derives from amyloid precursor protein (APP), a type I transmembrane protein involved in neural development and synaptic plasticity [11,12,13]. The amyloidogenic pathway begins with the β-secretase cleavage of APP, generating secreted N-terminal fragment (sAPPβ) and leaving APP C-terminal fragment β (C99) embedded in the membrane [14,15]. Then, γ-secretase is responsible for the cleavage of C99 and thus the production of cytosolic-AICD [16,17]. In addition, this initial cleavage and the subsequent trimming by γ-secretase generate Aβ45-49. Lastly, these long Aβ intermediates are further trimmed by γ-secretase within the membrane, generating and secreting Aβ peptides ranging from 37 to 43 amino acids (Aβ37-43) [18,19]. Early onset familial AD (FAD) is associated with a mutation in one of three genes: APP, presenilin-1 (PSEN1), and presenilin-2 (PSEN2) [20,21,22]. APP mutations generally appear near γ-secretase cleavage sites in the transmembrane portion, and presenilin is the catalytic component of the γ-secretase complex, suggesting that C99 processing by γ-secretase plays a central role in FAD pathogenesis.

Significant levels of Aβ are observed extracellularly. However, local enrichment post-production within neurons and/or internalization through receptor binding can lead to the intracellular accumulation of Aβ. We detected Aβ in late endosomes and lysosomes where γ-secretase predominantly cleaves C99 in intact neurons [23], indicating that Aβ is locally enriched post-generation within the neurons. A substantial number of studies suggest that intracellular Aβ build up may be part of early AD pathogenesis, appearing before the development of plaques, reviewed in [24]. Specifically, high Aβ concentrations can be found in the neurons in the hippocampus and entorhinal cortex of patients with mild cognitive impairment–regions of the brain particularly susceptible to neuropathology in AD [25,26]. In transgenic rodents designed to develop amyloidosis, large amounts of early intraneuronal Aβ were observed prior to the formation of plaques, which could be linked to deficits in synaptic plasticity, learning, and memory [27,28]. A recent study has added a new significant insight that the deficit in the autolysosome acidification, accompanied by the accumulation of C99 and Aβ peptides, is an early event in neurons, and such neurons could be the principal sources of neuritic plaques in amyloidosis models [29].

In this report, we further characterized the properties of intracellular Aβ by employing multiplexed immunocytochemistry and fluorescence lifetime imaging microscopy (FLIM) [30]. We verified in intact neurons that our immunostaining approach detects both Aβ intermediates bound to γ-secretase and Aβ peptides dissociated from the protease complex. Moreover, we uncovered that the dissociated Aβ binds to glycoproteins located on the inner membrane of lysosomes. Furthermore, we found that individual neurons express different levels of intracellular Aβ. Our study adds new information about the characteristics of intracellular Aβ, which could help elucidate the potentially important role(s) that C99 and Aβ may play in late endosomes and lysosomes, and potentially in autolysosomes within the neurons.

## 2. Materials and Methods

### 2.1. Antibodies and Reagent

Anti-Aβ 6E10 and 4G8 antibodies were purchased from BioLegend (San Diego, CA, USA), anti-APP C-terminus antibody was from Sigma-Aldrich (St. Louis, MO, USA), anti-Cathepsin B antibody was from Abcam (Cambridge, UK), anti-Galectin 3 antibody was from Abcam, anti-LAMP1 antibodies were from Sigma-Aldrich and Abcam, anti-nicastrin antibody was from Novus Biologicals (Littleton, CO, USA), and anti-V-ATPase antibody was from Abcam. Normal rabbit IgG antibody was purchased from Cell Signaling Technology, Inc. (Danvers, MA, USA), whilst Alexa488 and Cy3-conjugated donkey anti-mouse/rabbit IgG were from Thermo Fisher Scientific (Waltham, MA, USA) and Jackson ImmunoResearch Inc. (West Grove, PA, USA), respectively. DAPT, γ-secretase inhibitor, was purchased from Abcam. DMSO and Chloroquine were purchased from Sigma-Aldrich.

### 2.2. Primary Neuronal Culture

Primary neurons were prepared as described previously [31]. Briefly, neurons were dissociated from the cortex of both male and female CD1 mouse embryos (Charles River Laboratories (Wilmington, MA, USA)) using the Papain Dissociation System (Worthington Biochemical Corporation (Lakewood, NJ, USA)). The neurons were maintained in Neurobasal medium containing B27 supplement, GlutaMAX Supplement, and penicillin–streptomycin (Thermo Fisher Scientific). A pAAV2/8 vector packaging the C99 720-670 cDNA was developed by the University of Pennsylvania Gene Therapy Program vector core (2.56 × 10^13^ GC/mL), which was used for the neuronal expression of the C99 720-670 biosensor.

### 2.3. Immunocytochemistry

Primary neurons were fixed with 4% paraformaldehyde (VWR International (Radnor, PA, USA)), washed in DPBS (Thermo Fisher Scientific), then permeabilized using 0.1% Triton X-100 + 1.5% normal donkey serum (Jackson ImmunoResearch, Inc.). Then, the neurons were incubated overnight with primary antibodies and Alexa488- or Cy3-conjugated secondary antibodies for 1 h. Lastly, the slide was layered with a coverslip (Thermo Fisher Scientific) using Fluoromount-G Mounting Medium (Thermo Fisher Scientific).

### 2.4. Immunohistochemistry

Mouse cortex brain tissues from Tg2576 APP transgenic mice [32] and wild-type controls (35 μm thickness) were stored in 30% glycerol. The tissue slices were thawed on ice and washed in DPBS. The samples were then permeabilized and blocked in a TBS solution containing 0.25% Triton-X100 and 5% NDS (TBS-X solution). Next, the TBS-X solution containing primary antibodies was added and left to incubate overnight at 4 °C. The tissue was washed in DPBS, Dk-α-Ms-Alexa488 and Dk-α-Rb-Cy3 secondary antibodies were added in the TBS-X solution and incubated for 1 h. Finally, the tissue was washed in DPBS and mounted on a slide using Fluoromount-G Mounting Medium (Thermo Fisher Scientific).

### 2.5. Confocal Microscopy

An Olympus FV3000RS Confocal Laser Scanning Microscope (Tokyo, Japan) was used to image both primary neurons and mouse brain tissues. Lasers at 488 nm, 561 nm, and 640 nm were used to excite Alexa488, Cy3, and the C99 720-670 probe. The emitted fluorescence from Alexa488, Cy3, and C99 720-670 was detected within 500–530 nm, 565–590 nm, and 700–800 nm. The 10x/0.25NA, 40x/0.95NA, and 60x/1.30NA objectives were used for imaging. Pseudo-colored images corresponding to the fluorescent emission ratios were generated in MATLAB (The MathWorks (Natick, MA, USA)).

### 2.6. Fluorescence Lifetime Imaging Microscopy (FLIM)

An Olympus FV3000RS confocal microscope and x60 oil immersion objective were used for FLIM analysis. A mode-locked Chameleon Ti: Sapphire laser (Coherent Inc. (Santa Clara, CA, USA)) set at 850 nm was used to excite the Alexa488 donor fluorophore. The emitted fluorescence was collected using the ET525/50m-2p filter (Chroma Technology Corp (Bellows Falls, VT, USA)). A high-speed photomultiplier tube (MCP R3809; Hamamatsu (Hamamatsu, Japan)) and a time-correlated single-photon counting acquisition board (SPC-830; Becker and Hickl GmbH (Berlin, Germany)) were used to measure the lifetime of the donor fluorophore. SPC Image software (Becker and Hickl GmbH) was used to analyze the acquired FLIM data. The donor fluorophore’s average lifetime (t1) was measured in the absence of the acceptor fluorophore (FRET absent). In the presence of the acceptor fluorophore (Cy3), exciting the donor fluorophore results in reduced donor emission energy if the donor and acceptor are less than 5–10 nm apart (FRET present), shortening the donor fluorophore lifetime (t2).

### 2.7. Co-Immunoprecipitation and Western Blotting

Tg2576 mouse brains were lysed in RIPA buffer (Sigma-Aldrich) with protease and phosphatase inhibitors cocktail (Thermo Fisher Scientific). The lysate was incubated with the LAMP1 cytoplasmic or normal rabbit IgG antibodies, and then precipitated using Protein G Dynabeads (Thermo Fisher Scientific). Immunoprecipitated proteins were eluted in the elution buffer (50 mM Glycine pH2.8, LDS sample buffer, Reducing Agent; Thermo Fisher Scientific) by boiling for 3 min at 95 °C. Then, the eluted samples were subjected to SDS–PAGE on NuPAGE 12% Bis-Tris Protein gels (Thermo Fisher Scientific). The Bio-Rad Wet electroblotting system (Bio-Rad) was used to transfer proteins from the gels to nitrocellulose membranes (Thermo Fisher Scientific). The detection was performed by immunoblotting with specific primary and corresponding fluorophore conjugated secondary antibodies and their fluorescent signals were detected using the LI-COR Odyssey CLx scanner (LI-COR Biosciences (Lincoln, NE, USA)).

### 2.8. Statistical Analysis

GraphPad Prism version 9 (GraphPad Software, LLC, (San Diego, CA, USA)) was used to perform statistical analysis. The D’Agostino and Pearson omnibus normality test was used to examine the Gaussian distribution of the data and the variance equality. Then, the data were compared using unpaired Student’s t-test, Mann–Whitney U test, or one-way ANOVA with post hoc Tukey HSD.

## 3. Results

### 3.1. FLIM Evidences the Presence of Aβ Intermediates Bound to γ-Secretase and Aβ Dissociated from the Protease Complex in Intact Neurons

**Figure 1:**
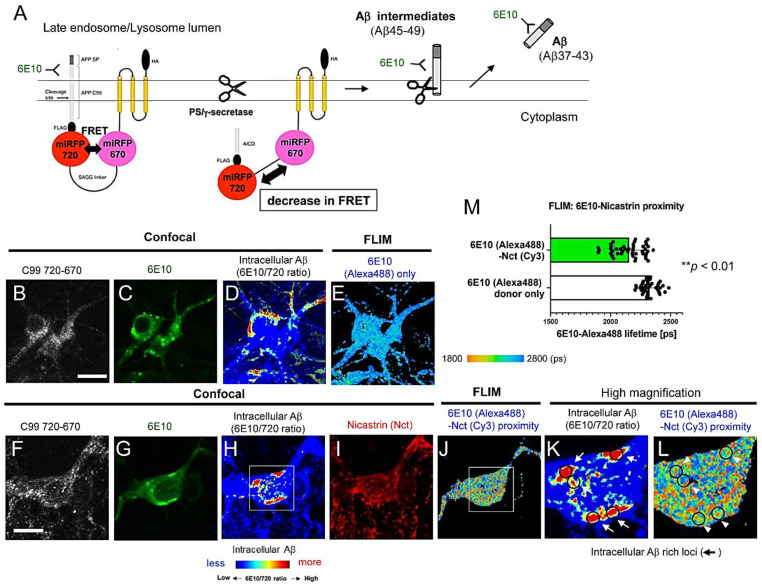
Both γ-secretase-bound Aβ intermediates and Aβ peptides dissociated from the protease complex are present in primary neurons expressing the C99 720-670 biosensor. (**A**) Schematic presentation of the C99 720-670 biosensor processing by γ-secretase. The initial cleavage of the C99 720-670 biosensor and subsequent trimming by γ-secretase generate Aβ45-49. These long Aβ intermediates are further trimmed by γ-secretase within the membrane, resulting in the generation and secretion of Aβ peptides (Aβ37-43) into the lumen of late endosomes and lysosomes. The 6E10 antibody, the epitope of which is on the N-terminus of human APP C99, captures the C99 720-670 biosensor, the longer Aβ intermediates, and the dissociated shorter Aβ species generated from the C99 720-670 biosensor. (**B**–**M**) Fluorescence lifetime imaging microscopy (FLIM) analysis of 6E10-Alexa488 in neurons expressing the C99 720-670 biosensor co-stained with either normal rabbit IgG-Cy3 (**B**–**E**) or nicastrin (Nct)-Cy3 antibodies (**F**–**L**). (**D**,**H**) The pseudo-color images of 6E10 over C99 720-670 emission ratio highlight the subcellular areas with increased intracellular Aβ (red = more Aβ). (**K**,**L**) show high magnification images corresponding to the squares in (**H**,**J**). Scale bar: 25 μm. (**M**) Quantification of FLIM analysis between 6E10-Alexa488 and Nct-Cy3 or rabbit normal IgG-Cy3 antibodies (donor only; FLIM negative control) in the intracellular Aβ-rich compartments (38–45 ROIs from 4–6 neurons, ** *p* < 0.01).

The selective detection of intracellular Aβ is challenging because antibodies against Aβ cross-react with the Aβ precursor fragments. Our recently developed genetically encoded Förster resonance energy transfer (FRET)-based C99 720-670 biosensor [23,33,34] has enabled us to uncover that C99 is predominantly processed by endogenous γ-secretase in late endosomes and lysosomes in intact/live neurons [23]. To develop the C99 720-670 biosensor, we first tagged the human C99 (APP-CTFβ) containing the APP signal peptide sequence with miRFP720 [35]. Then, miRFP670 [36] was connected to the miRFP720 with 80 amino acids (a.a.) SAGG-repeat linker [37]. Lastly, the miRFP670 was stabilized near the membrane by fusion to the transmembrane anchoring domain (Figure 1A) [33]. The cleavage of APP C99 within the C99 720-670 biosensor by endogenous γ-secretase results in the production of Aβ intermediates and APP intracellular domain 720-670 (AICD 720-670) (Figure 1A). This results in a change in the proximity and/or orientation between the miRFP670 and miRFP720, which we record by ratiometric spectral FRET analysis as a reduction in the FRET efficiency (Figure 1A) [33]. Furthermore, neurons expressing the C99 720-670 biosensor were stained with the Aβ 6E10 antibody (recognizes human Aβ 1-16), detecting the C99 720-670 biosensor as well as the biosensor-derived intracellular Aβ (Figure 1A) [23]. Moreover, when the intensity of the 6E10-Alexa488 emission was divided by that of the C99 720-670 biosensor, we found that the 6E10 over the C99 720-670 ratio was significantly decreased by treatment with DAPT, a potent γ-secretase inhibitor [23]. Since the 6E10/C99 720-670 ratio can be color-coded and mapped over the cell, this novel multiplexed immunostaining approach permits us to visualize the cell compartment(s) with increased 6E10 Alexa488/C99 720-670 ratios as sites where intracellular Aβ is enriched within the neurons. Notably, we reported that intracellular Aβ is significantly increased in the late endosomes and lysosomes, aligning with exactly where the C99 720-670 biosensor is predominantly processed by γ-secretase, and suggesting that γ-secretase-mediated C99 processing results in the localized enrichment of Aβ within the neurons [23].

To determine the detailed properties of intracellular Aβ, we first stained neurons expressing the C99 720-670 biosensor with 6E10-Alexa488 and nicastrin (Nct; one of the components of γ-secretase)-Cy3 (Figure 1F–L) or normal rabbit IgG-Cy3 antibodies (FLIM negative control) (Figure 1B–E). Then, confocal microscopy was performed to identify the subcellular areas with higher intracellular Aβ levels (Figure 1D,H,K). Lastly, FLIM was employed to measure the lifetime of 6E10-Alexa488 in the Aβ-rich compartments (Figure 1E,J,L). FLIM monitors the lifetimes of the donor fluorophore as a measure of proximity to an acceptor fluorophore, with shorter lifetimes reflective of the energy transfer from the donor to acceptor fluorophores (from Alexa488-6E10 to Cy3-Nct/γ-secretase in this case) due to their closer proximity [30]. Therefore, the shorter 6E10-Alexa488 lifetime due to FRET between 6E10-Alexa488 and Nct-Cy3 indicates the presence of Aβ intermediates (i.e., Aβ45-49) bound to γ-secretase in the cells. On the other hand, the longer 6E10-Alexa488 lifetime indicates that the subcellular compartments predominantly contain Aβ37-43 species dissociated from the protease complex (Figure 1A). Indeed, we found that several regions of interest (ROIs) displayed significantly decreased 6E10-Alexa488 lifetimes, while others showed similar lifetimes to that of neurons only stained with 6E10-Alexa488 (a negative control of FRET) (Figure 1E,J,L,M). These results suggest that, as expected, the 6E10 antibody captures Aβ peptides dissociated from the γ-secretase complex and secreted into the lumen of late endosomes and lysosomes. Moreover, the antibody also detects γ-secretase-bound Aβ intermediates within the membrane and these short and long Aβ species are heterogeneously expressed inside the neurons.

### 3.2. Aβ Is Bound to Glycoproteins on the Inner Membrane of Lysosomes

**Figure 2:**
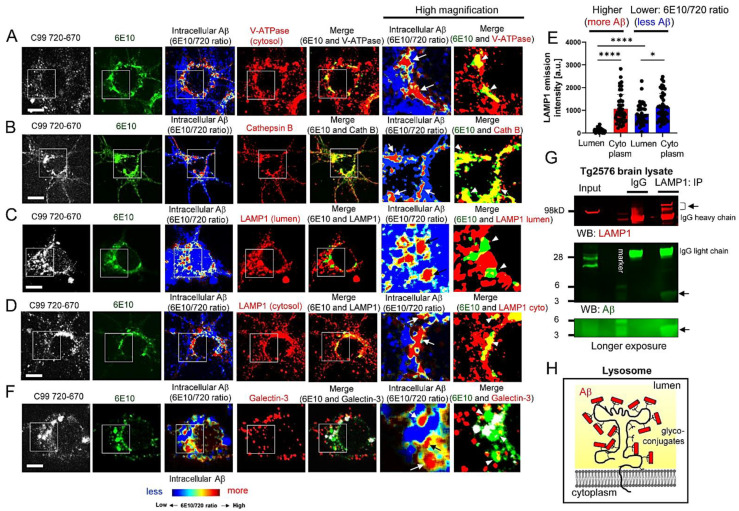
Aβ is bound to glycoproteins on the inner leaflet of lysosome membrane. (**A**) Neurons expressing the C99 720-670 biosensor were co-stained with 6E10-Alexa488 and Cy3-labeled V-ATPase, (**B**) Cathepsin B, (**C**) LAMP1 (lumen epitope), or (**D**) LAMP1 (cytoplasmic epitope). Scale bar: 10 μm. (**E**) LAMP1-Cy3 emission intensity was measured in the ROIs with high or low 6E10 Alexa488/C99 720-670 ratios (44–50 ROIs from 5–8 neurons, * *p* < 0.05, **** *p* < 0.0001). Arbitrary unit: a.u. (**F**) Post-treatment with 50 µM chloroquine for 2 h to induce lysosome membrane rupture, the neurons were co-stained with Alexa488-6E10 and Cy3-Galectin 3. Scale bar: 10 μm. (**G**) Tg2576 mouse brain lysate was immunoprecipitated with the LAMP1 cytoplasmic or normal IgG antibodies, followed by Western blotting using Aβ 6E10 and 4G8 antibodies. (**H**) Schematic presentation: Aβ binds to the glycoconjugates of lysosomal proteins located on the inner leaflet.

We previously showed that subcellular compartments with higher 6E10 Alexa488/C99 720-670 ratios (i.e., intracellular Aβ-rich areas) are immunopositive with Rab7 (late endosomes) and LAMP1 (lysosomes) antibodies [23]. To further verify the enrichment of Aβ in late endosomes and lysosomes, neurons expressing the C99 720-670 biosensor were stained with an antibody against Vacuolar-type ATPase (V-ATPase), a proton pump essential for the late endosome and lysosome acidification [38]. We detected the V-ATPase fluorescence signal in the Aβ-rich areas (Figure 2A). Furthermore, neurons expressing the C99 720-670 biosensor were stained with an antibody against Cathepsin B, a lysosomal hydrolase. We also found that the Aβ-rich areas display strong Cathepsin B positivity (Figure 2B), further evidencing that Aβ is localized within the acidic compartments in intact neurons.

Surprisingly, we found that the immunoreactivity of a LAMP1 antibody, the epitope of which localizes in the lumen of lysosomes, was significantly decreased in the intracellular Aβ-rich areas (Figure 2C). On the other hand, the fluorescence signal from a LAMP1 antibody detecting the cytoplasmic epitope overlaps with that from intracellular Aβ (Figure 2D). Quantitative analysis shows that the fluorescence from the lumen antibody was significantly weaker than that from the LAMP1 cytoplasmic antibody in the Aβ-rich ROIs. On the other hand, the sites with low 6E10 Alexa488/C99 720-670 ratios (i.e., C99-rich) did not display such an extensive difference between the two antibodies’ fluorescence (Figure 2E). These results suggest that Aβ interferes with the lumen epitope of the LAMP1 antibody and may bind to the glycoproteins expressed on the inner membrane of lysosomes.

To further validate this scenario, neurons expressing the C99 720-670 biosensor were treated with 50 µM Chloroquine for 2 h to induce lysosomal membrane rupture [39], followed by immunostaining with a Galectin-3 antibody. Galectin-3 is normally expressed in the cytoplasm; however, once the lysosomal membrane is permeabilized, Galectin-3 localizes inside the lysosomes and recognizes galactose-containing glycoconjugates on glycoproteins integral to the inner membrane of lysosomes [40]. Strikingly, we found that the Aβ-rich lysosomes do not co-localize with the Galectin-3 puncta (Figure 2F), suggesting that the binding of Galectin-3 to glycoconjugates is interfered with by Aβ.

Furthermore, we performed co-immunoprecipitation to ensure the interaction between Aβ and LAMP1. Tg2576 APP transgenic mouse [32] brain lysate was immunoprecipitated with the LAMP1 cytoplasmic antibody, followed by Western blotting using Aβ 6E10 and 4G8 antibodies. As a result, we detected Aβ in the LAMP1-precipitates (Figure 2G), further evidencing the binding of Aβ to the lumen region of LAMP1. Collectively, these results suggest that Aβ binds to glycoproteins on the inner membrane of lysosomes (Figure 2H).

### 3.3. Individual Neurons Contain Different Levels of Intracellular Aβ

**Figure 3:**
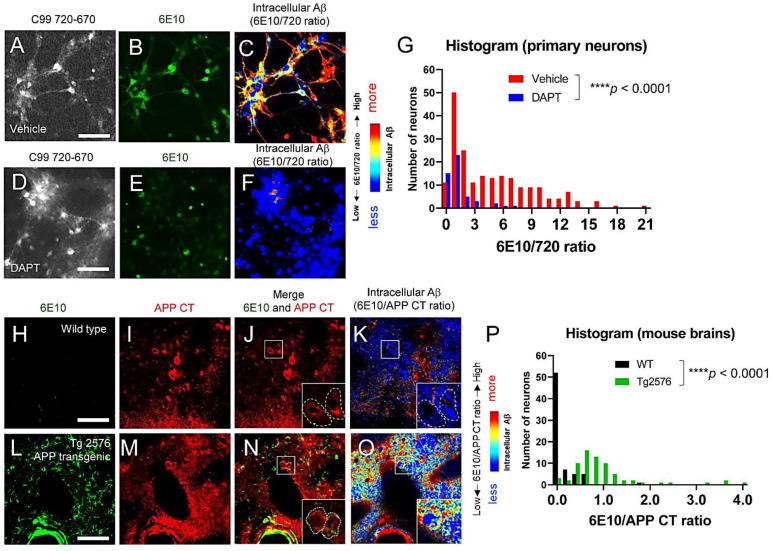
Cell-to-cell heterogeneity in intracellular Aβ levels. (**A**–**G**) Neurons expressing the C99 720-670 biosensor were treated either with 1 µM DAPT (**D**–**F**) or vehicle control (**A**–**C**) for 16 h, followed by staining with Alexa488-6E10. The pseudo-color images of Alexa488-6E10 over C99 720-670 emission ratio (red = more Aβ) (**C**, **F**) and quantification (**G**) illustrate that intracellular Aβ levels are distinct among neurons (50 neurons, **** *p* < 0.0001). Scale bar: 50 μm. (**H**–**P**) Brain sections from Tg2576 APP transgenic (**L**–**O**) or wild-type control mice (**H**–**K**) were stained with Alexa488-6E10 and Cy3-APP C-term antibodies. As expected, the Alexa488-6E10 over Cy3-APP C-term emission ratio is significantly higher in Tg2576 than in wild-type brain sections, and individual cells in the Tg2576 brain exhibit distinct levels of Alexa488-6E10/Cy3-APP CT ratio (70 cells from 3 independent mice, **** *p* < 0.0001) (**P**). Scale bar: 40 μm.

It was reported that individual neurons produce and secrete different levels of Aβ in the conditioned medium [41]. Similarly, we showed that C99 processing by γ-secretase is heterogeneously regulated on a cell-by-cell basis in live neurons [31]. Therefore, we examined whether individual neurons expressed different levels of intracellular Aβ. As such, primary neurons expressing the C99 720-670 biosensor were stained with Alexa488-6E10 antibody, and the 6E10-Alexa488/C99 720-670 ratios were measured in neurons on a cell-by-cell basis (Figure 3A–F). We uncovered that the 6E10-Alexa488/C99 720-670 ratios vary among individual neurons, and the wide distribution of the ratios is “canceled” by the treatment with a γ-secretase inhibitor: DAPT (1 µM for 16 h) (Figure 3G). Furthermore, to corroborate the physiological relevance of the findings from primary neurons, the brain tissues from Tg2576 APP transgenic mice were stained with 6E10 (Alexa488) and APP C-terminus (CT) (Cy3) antibodies. To quantitatively assess intracellular Aβ in the neurons of Tg2576 mice, we calculated the ratio of 6E10-Alexa488 (i.e., Aβ + APP/C99) over APP CT-Cy3 (i.e., APP/C99 only) fluorescence intensity on a cell-by-cell basis. We found that, as expected, the 6E10-Alexa488/APP CT-Cy3 ratios are significantly higher in the Tg2576 tissues than in wild-type littermate controls (Figure 3H–O) and that individual neurons display different 6E10-Alexa488/APP CT-Cy3 ratios (Figure 3P). These results suggest that individual neurons contain different levels of intracellular Aβ both in cultured neurons and in mouse brains in vivo.

## 4. Discussion

Numerous mutations causing FAD were identified on the genes encoding APP, PSEN1, and PSEN2 (https://www.alzforum.org/mutations: accessed on 19 August 2022), highlighting the great importance of APP processing by γ-secretase in pathogenesis. Aβ peptides are generated from the sequential cleavage of APP by β- and γ-secretases within vesicles inside cells and on the plasma membrane, and are normally secreted under physiological conditions [42]. On the other hand, there is evidence showing the presence of intracellular Aβ, particularly in mouse models of amyloidosis [24,25,26,27,28,29], suggesting that some Aβ is retained inside the neurons. However, the detailed properties of intracellular Aβ are not fully established. We have recently developed APP C99-based FRET biosensors that enable the quantitative recording of C99 processing by γ-secretase in live neurons [23,31,33,34]. Combining these novel reporter probes with high-resolution confocal microscopy, we successfully detected predominant γ-secretase activity in late endosomes and lysosomes in intact neurons [23]. Furthermore, we uncovered that Aβ peptides derived from the FRET sensor were significantly enriched in the same subcellular loci [23]. In this new study, we employed FLIM and multiplexing immunocytochemistry to better understand the characteristics of Aβ enriched in the acidic compartments. Here, we provide evidence that: 1) not only shorter Aβ dissociated from the γ-secretase complex but also long Aβ intermediates that are bound to the γ-secretase complex are present in intact neurons (Figure 1); 2) Aβ is associated with glycoproteins on the inner leaflet of the lysosomal membrane (Figure 2); and furthermore, we show that 3) intracellular Aβ levels are heterogeneously regulated on a cell-by-cell basis in primary neurons and APP transgenic mouse brains (Figure 3).

One of the critical issues in the specific detection of intracellular Aβ is that many anti-Aβ antibodies capture not only intracellular Aβ but also the Aβ sequence embedded in the APP holoprotein and C99 fragment. However, several studies compared the patterns of immunoreactivity for antibodies against Aβ and APP C-terminal fragments (APP CTFs) and found that Aβ and APP-CTFs exhibit qualitatively distinct immunoreactivities in different subcellular localization and varied temporal expression [26,43,44]. In addition, we recently developed a new protocol combining immunocytochemical detection with a ratiometric FRET-based approach using the C99 720-670 probe, which allows the quantitative recording of the C99 cleavage by γ-secretase and the qualitative, yet relatively quantitative, detection of intracellular Aβ [23]. Furthermore, the unique spectral properties and thus the multiplexing capability of NIR fluorescent proteins such as miRFP720 have permitted us not only to visualize intracellular Aβ but also to address several unsolved questions, such as which Aβ species reside within neurons and exactly where intracellular Aβ is located. Our FLIM analysis using a nicastrin antibody, detecting the γ-secretase complex and facing its epitope toward C99/long Aβ, provides evidence that long Aβ intermediates bound to γ-secretase exist within the neurons (Figure 1). The existence and quantity of shorter Aβ peptides (Aβ37-43) are well validated due to the availability of specific antibodies, whereas detecting long Aβ intermediates remains challenging since their C-terminus region is embedded within the membrane. Biochemical assays employing urea-PAGE and Western blotting or liquid chromatography coupled to tandem mass spectrometry (LC-MS/MS) have been used to determine the presence of long Aβ intermediates in cell/tissue lysates and/or isolated enzyme assays [18,19,45,46,47]. Our FLIM analysis adds new evidence supporting the presence of γ-secretase-bound long Aβ intermediates in intact cells, representing a new imaging platform to determine the effect of PSEN and APP mutations or pharmacological agents on long Aβ-γ-secretase complexes. Importantly, a recent study reported that FAD-causing APP mutations significantly increase the levels of long Aβ intermediates [47].

To further ensure local Aβ enrichment in neuronal late endosomes and lysosomes, this study used V-ATPase and Cathepsin B antibodies (Figure 2) in addition to Rab7 and LAMP1 antibodies in our initial study [23]. Surprisingly, detection with antibodies to the lumen site of LAMP1 and Galectin-3 was mostly abolished in Aβ (+) compartments (Figure 2), suggesting that Aβ may associate with the glycoconjugates of lysosomal proteins on the inner membrane and thus prevent the antibodies binding. Interestingly, while several reports demonstrate that Aβ causes lysosomal membrane rupture [48,49,50], we found that Aβ (+) lysosomes are Cathepsin B positive (Figure 2). This suggests that the Aβ we detected in lysosomes may not (or not yet) cause membrane permeabilization. This discrepancy between the previous and our studies could be either because of the difference between the internalization of synthetic Aβ peptides (previous studies) and the local enrichment of Aβ post-production within neurons (ours), or the different concentration and time of Aβ in lysosomes. It would be interesting to determine whether the Aβ retained in lysosomes post-production causes lysosomal membrane rupture or plays a protective role to prevent the leakage of lysosomal components into the cytoplasm, since it could be linked to the propagation and cell invasion of misfolded proteins such as tau and alpha-synuclein [51,52].

It has very recently been reported that C99 and Aβ are significantly accumulated in poorly acidified autolysosomes, resulting in the formation of large membrane blebs containing autophagic vacuoles filled with C99/Aβ inside neurons. Importantly, these unique blebs seed Aβ fibrils within the neurons, which could be the source of neuritic plaques in amyloidosis mouse models [29]. This study suggests that monomeric Aβ is converted into aggregated forms rich in β-sheet structures within autolysosomes; however, the mechanism(s) by which Aβ is aggregated within the lysosomal/autophagic vesicles remains unclear. On the plasma membrane, it is well validated that Aβ binds to monosialoganglioside GM1 [53]. Upon binding, Aβ changes its conformation from a random coil to a-helix and then β-sheet rich structures dependent on peptide densities [54,55]. We detected Aβ ~4kD in size in the LAMP1 precipitates (Figure 2), suggesting that Aβ monomer and/or oligomers (that are monomialized by LDS) bind to LAMP1 but Aβ fibrils do not. In this regard, it would be interesting to examine whether the binding of Aβ to glycoproteins on the inner membrane of lysosomes is a critical step to seed Aβ fibrils within the autolysosomes.

## 5. Conclusions

Our new study demonstrates the presence of long Aβ intermediates bound to γ-secretase and Aβ peptides dissociated from the protease complex in intact neurons (Figure 1). Surprisingly, the dissociated Aβ is bound to the glycoproteins on the inner membrane of lysosomes (Figure 2). Furthermore, we show that individual neurons contain different levels of intracellular Aβ in primary cultures and APP transgenic mouse brains (Figure 3). Cell-to-cell heterogeneity in endogenous γ-secretase activity [31,41] could partly explain the different levels of intracellular Aβ. Our future studies will address several remaining questions that include exactly which Aβ species are enriched in the late endosomes and lysosomes and, importantly, whether the Aβ in endo-lysosomes is relevant to AD. A better understanding of the function(s) of endo-lysosomal Aβ would provide significant insight into the molecular mechanism underlying AD neurodegeneration.

## Data Availability

Not applicable.
